# Breakpoint characterization of large deletions in *EXT1 *or *EXT2 *in 10 Multiple Osteochondromas families

**DOI:** 10.1186/1471-2350-12-85

**Published:** 2011-06-26

**Authors:** Ivy Jennes, Danielle de Jong, Kirsten Mees, Pancras CW Hogendoorn, Karoly Szuhai, Wim Wuyts

**Affiliations:** 1 Department of Medical Genetics, University and University Hospital of Antwerp, 2650 Edegem, Belgium; 2 Department of Molecular Cell Biology, Leiden University Medical Center, 2333 ZA Leiden, The Netherlands; 3 Department of Pathology, Leiden University Medical Center, 2333 ZA Leiden, The Netherlands

**Keywords:** Multiple osteochondromas, *EXT1, EXT2*, deletion breakpoint, arrayCGH, NAHR, NHEJ, MMRDR, bone neoplasm

## Abstract

**Background:**

Osteochondromas (cartilage-capped bone tumors) are by far the most commonly treated of all primary benign bone tumors (50%). In 15% of cases, these tumors occur in the context of a hereditary syndrome called multiple osteochondromas (MO), an autosomal dominant skeletal disorder characterized by the formation of multiple cartilage-capped bone tumors at children's metaphyses. MO is caused by various mutations in *EXT1 *or *EXT2*, whereby large genomic deletions (single-or multi-exonic) are responsible for up to 8% of MO-cases.

**Methods:**

Here we report on the first molecular characterization of ten large *EXT1*- and *EXT2*-deletions in MO-patients. Deletions were initially indentified using MLPA or FISH analysis and were subsequently characterized using an MO-specific tiling path array, allele-specific PCR-amplification and sequencing analysis.

**Results:**

Within the set of ten large deletions, the deleted regions ranged from 2.7 to 260 kb. One *EXT2 *exon 8 deletion was found to be recurrent. All breakpoints were located outside the coding exons of *EXT1 *and *EXT2*. Non-allelic homologous recombination (NAHR) mediated by *Alu*-sequences, microhomology mediated replication dependent recombination (MMRDR) and non-homologous end-joining (NHEJ) were hypothesized as the causal mechanisms in different deletions.

**Conclusions:**

Molecular characterization of *EXT1*- and *EXT2*-deletion breakpoints in MO-patients indicates that NAHR between *Alu-*sequences as well as NHEJ are causal and that the majority of these deletions are nonrecurring. These observations emphasize once more the huge genetic variability which is characteristic for MO. To our knowledge, this is the first study characterizing large genomic deletions in *EXT1 *and *EXT2*.

## Background

Osteochondromas (cartilage-capped bone tumors) are by far the most commonly treated of all primary benign bone tumors (50%) [1]. Most osteochondromas appear as solitary, nonhereditary lesions, but in 15% of cases these tumors occur as multiple lesions in the context of multiple osteochondromas (MO) [2] (OMIM 133700-133701), previously known as osteocartilaginous exostosis or multiple hereditary exostosis (MHE/HME). The prevalence of this autosomal dominant skeletal disorder is estimated to be 1/50.000 in the Western population [3]. MO is characterized by the formation of multiple osteochondromas mainly arising from the growth plate area in the juxta-epiphyseal region of long tubular bones. These bone neoplasmas are caused by an increased chondrocyte proliferation and bone growth at children's metaphyses [4]. During the first decades of life, they develop gradually and grow in size and number, until skeletal maturation is achieved at the end of puberty with the closing of the growth plates [5]. MO is characterized by a significant inter-and intrafamilial phenotypic variability, including variation in the number and size of osteochondromas, the number and location of involved bones, and the degree of the deformities. Various complications arise from these benign tumors, but malignant transformation towards a chondrosarcoma is by far the most serious one, occurring in 0.5-2% of patients [2,3,6].

MO is caused by mutations in *Exostosin*-1 (*EXT1*) (OMIM *608177) [7] or *Exostosin*-2 (*EXT2*) (OMIM *608210) [8,9]. *EXT1 *consists of 11 exons, is located at 8q24.11-q24.13 and spans ~350 kb [10], while *EXT2*, located at 11p12-p11, comprises of 16 exons and spanning ~108 kb [11]. Both genes act as tumor suppressor genes that belong to the EXT multigene family [12] and are ubiquitously expressed. All members of this family encode proteins that are involved in the adhesion and/or polymerization of heparin sulphate (HS) chains at HS proteoglycans (HSPG's). The HSPG's play a crucial role in the diffusion of Indian Hedgehog (Ihh), which is important for the regulation of chondrocyte proliferation and differentiation. In osteochondromas, the cartilage cells of the tumor cap are heterogeneous regarding to the mutation status in *EXT1 *or *EXT2*, with a mixture of homozygous and heterozygous *EXT*-inactivated cells [13].

MO is characterized by a huge genetic heterogeneity. The online Multiple Osteochondromas Mutation Database (MOdb) http://medgen.ua.ac.be/LOVDv.2.0/home.php provides an overview on all currently reported MO-causing mutations. To date, the database contains more than 1000 MO-patients representing more than 600 unique *EXT1*- or *EXT2*-mutations. In 3.7% of the MOdb-cases, the disease is caused by a large deletion consisting of at least one *EXT1- *or *EXT2*-exon [14]. However, these rearrangements are expected to account for up to 5-8% of MO cases [15,16].

Large genomic deletions can occur during the repair of double strand breaks (DSB's) in DNA by several mechanisms [17], including non-homologous end-joining (NHEJ) (including classical-and non-classical NHEJ), microhomology-mediated replication-dependent recombination (MMRDR) and homologous recombination (HR) (including non-allelic homologous recombination (NAHR) and single strand annealing (SSA)). Large deletions containing *EXT1 *or *EXT2 *can result in contiguous gene syndromes. The Tricho-Rhino-Phalangeal syndrome (TRPSII), also known as the Langer-Giedion syndrome (LGS), is caused by deletions in 8q24.11-q24.13, including the *EXT1 *and *TRPS1 *genes, and is characterized by patients having MO and TRPSI characteristics (facial dysmorphy, microcephaly, sparse scalp hair, short stature and cone-shaped epiphyses) as well as mental retardation [18,19]. The Potocki-Shaffer syndrome (P11pDs or DEFECT11 syndrome) (OMIM 601224) is caused by deletions in 11p11.2-p12, including the *EXT2 *and *ALX4 *genes, and is characterized by patients having MO combined with Foramina Parietalia Permagna (FPP) (OMIM 168500), mental retardation and craniofacial dysotosis [20,21].

Smaller single or multi-exon rearrangements in *EXT1 *or *EXT2 *cause a phenotype of multiple osteochondromas only. They are routinely screened for by Multiplex Ligation-dependent Probe Amplification (MLPA) [16]. However, this diagnostic technique does not provide detailed information on the breakpoints, so different deletions involving the same exon(s) cannot be distinguished, nor can insight be acquired into the developmental mechanism of these gross rearrangements. Therefore, we characterized for the first time the MO-causing genomic deletions in *EXT1 *or *EXT2 *in index patients from ten unrelated families, using an MO-specific tiling path array, allele-specific PCR-amplification and direct sequencing.

## Methods

### Patients

This study included index patients from ten unrelated families (Family 89, 122, 150, 200, 250, 279, 300, 334, 338 and 361) originating from all over Europe and the USA. The study was approved by the ethical committee of the University of Antwerp under number A04-64. Consent of the patients was obtained. All patients had radiological confirmed multiple osteochondromas. Genomic DNA was isolated from peripheral blood according to standard procedures. Two patients were reported to be sporadic cases while for six patients the disease was reported to be inherited. Details on patients are listed in Table 1.

**Table 1:** Overview on the characteristics of 10 MO-families

Family number	Patient number	Disease occurrence	Geographic origin	Deletion at exon-level	Forward PCR-primer	Reverse PCR-primer
**EXT1**						

89	54782	Familial	USA	exon 2-11	GGGCAAAATTGTCCTCTGTC	TTGGTTGAGAGCCCAGATTT

200	73585	Familial	Spain	exon 8	CCCCACACACACACACTACA	TCAAATGCATAAACTCACTTCTGA

250	74559	Familial	USA	exon 2-3	CGGGAGAGAGAAACCATGAA	TGAGAGGGGAAAAACACCAG

300	86255	Familial	-	exon 6-7	CCAAACCTGTTATGGGAACC	GATTTTCCCCCAGATGGTGT

338	91636	De novo	Bulgaria	exon 11	TCATTATGTGGTGCATGACTG	CCTTTATGAAAGGCCACCAG

361	94668	-	Denmark	exon 2-11	GTTGACTGGTCCCACTGGTT	TGTCTTCCCAATCCTGTTTCA

**EXT2**						

122	59931	Familial	Spain	exon 8	CCCATTGCCTTTGCATTACT	TGACTCCTCATGCAACCAAA

334	91391	De novo	Bulgaria	exon 8	CCCATTGCCTTTGCATTACT	TGACTCCTCATGCAACCAAA

150	60859	Familial	USA	exon 2	ATGCAGGATGCCAAAATA	CCCAACAGCACATCAGACAC

279	84389	Familial	Macedonia	exon 8	GGATGGAAATGTGGGATAAGG	CACACCACCAGGGTTAATGC

Overview on the disease occurrence, the geographic origin as well as the deletion at exon-level. Patient numbers were provided together with MO-family numbers, so additional information on our patients can be accessed in the MOdb http://medgen.ua.ac.be/LOVDv.2.0/home.php. Additionally, primers for allele-specific PCR-amplification of the deletion breakpoint are listed.

All patients were selected from a larger cohort of MO patients based on the presence of a single or multi exon deletion in the EXT1 or EXT2 gene, detected by MLPA analysis [16] or FISH analysis with *EXT1*-probes 46F10, 65G5 and 90D8 [7] or *EXT2*-probes A1151 and D0694 [9]. The MLPA/FISH findings on two of these families (Families 150 and 200) have been reported previously [16].

### ArrayCGH tiling path array

High-resolution oligonucleotide arrayCGH analysis was performed using a custom-made Agilent oligonucleotide-based chip (Agilent Technologies, Santa Clara, CA, USA) in accordance with manufacturers' instructions. This array contains ~44.000 probes and has a tiling coverage for *EXT1 *and *EXT2*, additional genes involved in HS-pathways and adjacent genes. This MO-specific tiling path array has been thoroughly described in [22].

### PCR-amplification & sequencing

For the exact mapping of the deletion breakpoints, allele specific PCR's were designed around the deletion breakpoints that had been mapped using arrayCGH. PCR-amplifications were performed by with a Touch Down temperature protocol using Platinum Taq DNA Polymerase (Invitrogen, San Diego, CA, USA) and the Advantage UltraPure dNTP Combination Kit (Clontech, Mountain View, CA, USA). Amplification products were subsequently sequenced using Big Dye Terminator Cycle Sequencing kit with sequencing analysis on an ABI3130xl genetic analyzer (Applied Biosystems, Foster City, CA, USA). Primers for PCR-amplification and sequencing analysis of breakpoints are listed in Table 1.

### In silico analysis of breakpoint regions

Sequences located 25 bp down-and upstream from the breakpoints as well as the breakpoint region were analyzed for the presence of DNA-motifs (and their complements) that are known to be associated with genomic deletions, being translin target sites (ATGCAG, GCCCWSSW), immunoglobulin heavy chain class switch repeats (GAGCT, GGGCT, GGGGT, TGGGG, TGAGC) and the deletion hotspot consensus sequence (TGRRKM) [23]. The same regions were also analyzed for purine-pyrimidine repeats. Finally, sequences 125 bp down-and upstream from the breakpoint junctions were analyzed for their AT-content.

Regions of 1 kb down-and upstream from the breakpoint junctions were additionally analyzed for repeats (*Alu*-elements and LINE's) with the UCSC human genome browser http://genome.ucsc.edu.

In the search for homologous sequences, alignment of sequences up to 10 kb down-and upstream from the breakpoint junctions was performed using the NCBI BLAST2-software http://www.ncbi.nlm.nih.gov/blast/bl2seq/wblast2.cgi.

## Results

### Identification of deletion breakpoints with ArrayCGH, PCR-amplification & sequencing

**Figure 1:**
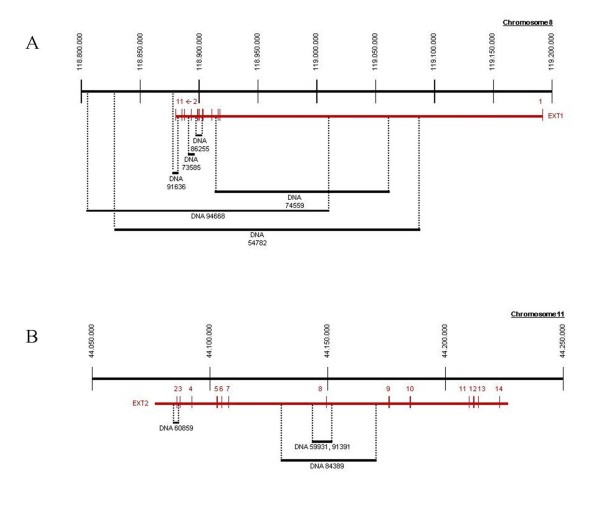
**To scale overview on the extend of all analyzed deletions**. A.) Deletions in EXT1, B.) Deletions in EXT2.

We subjected samples from the ten index patients with heterozygous causal *EXT1 *or *EXT2 *deletions to tiling path arrayCGH, allele-specific PCR-amplification and sequencing analysis for characterization of the deletion breakpoints. This allowed determination of the precise size of the respective deletions and the position of the proximal and distal breakpoints or breakpoint regions for all ten patients. An overall view to scale on the extend of all analyzed *EXT1 *and *EXT2 *deletions is provided in Figure 1, while Table 2 summarizes sequences flanking the deletions breakpoints.

**Table 2:** Overview on the characteristics of the deletions

Gene	Family number	Deletion size (bp)	Position breakpoints	Breakpoint sequences	DNA sequence motifs
EXT1	89	259.450	Proximal: 118.829.422-118.829.425 Distal: 119.088.871-119.088.874	ATTTTCCTTGAAAGGAGGCCTCTAGTTTTC|accaagttatcaaaaa**tattca**aga-- --catcttccacccatttggatggattttttc|CTGTTCTCAGAAGCTGGGTTTGACC	TATTCA: Deletion hotspot consensus sequence (complement)

EXT1	200	5885	Proximal: 118.891.184-118.891.186 Distal: 118.897.068-118.897.070	ACAGGCGGGAGCCACTGTACCTGGCCAA|cattgttgttcgttttaaaggagtt-- --ggtgtaggtaatcacaacctaatttcaa|TTTTCTAGCAGAT**GCTCA**CCATCCC	GCTCA: Immuno heavy chain class swith repeat (complement)

EXT1	250	148.254	Proximal: 118.914.819 - 118.914.854 Distal: 119.063.074-119.063.109	TTTTTTTAGTAGAAC**TGGGGT**TTCGCCATGTTGGCCAGGCTGGTCTTGAACTCCTGACCTC| aagtgatctgcctgccttggcctcc-- --tatttttagtagagatggtgtttcaccatgttggccaggctggtcttgaactcctgacctc|GT**GATCCA**CCT**GCCTCA**GCCTCCCA	TGGGG: Immuno heavy chain class swith repeat GGGGT: Immuno heavy chain class swith repeat GATCCA: Deletion hotspot consensus sequence (complement) GCCTCA: Deletion hotspot consensus sequence (complement)

EXT1	300	6176	Proximal: 118.896.107-118.896.110 Distal: 118.902.282-118.902.285	CTCACATACTTTTTTTCTCAGCTATATCA|ctgctacacgaagaagagattctgg-- --caggtgtgaattcagagaggatgtcatca |TCCTTACTATAACTTCTGGAAGAAG	-

EXT1	338	5688	Proximal: 118.877.923 - 118.877.925 Distal: 118.883.610-118.883.612	ATTCTACCAAACAGTATTTCTAGTAATT|catacatctttaacaaaaaaatcta-- --attacagtaggctatgttagcctttatt| TTGGTGGTTCTCAAATACCTGGTGA	-

EXT1	361	205.798	Proximal: 118.805.191 - 118.805.193 Distal: 119.010.988-119.010.990	AAATCTAT**TGAGC**CTGCTTATGATTCTTT|ggttttggagg**aggagggc**actaat-- --ttgttttgttttgttttgttttttgcttt| TCTGAGATGGAGTTTTGCTCTCGTT	TGAGC: Immuno heavy chain class swith repeat AGGAGGGC: Translin binding site (complement)

EXT2	122, 334	8690	Proximal: 44.143.251-44.143.282 Distal: 44.151.940 - 44.151.971	GATCTCCTGACCTCGTGATCCGCCCGCCTCGGCCTCCCAAAGTGCTGGGATTACAGG| cg**tgagc**caccgcgcccggcccaca-- --catctcctgatcttgtgatccgcctgcctcggcctcccaaagtgctgggattacagg|TG**TGAGC**CACCGCGCCCGGCCTTTT	TGAGC: Immuno heavy chain class swith repeat TGAGC: Immuno heavy chain class swith repeat

EXT2	150	2749	Proximal: 44.084.367-44.084.368 Distal: 44.087.115 - 44.087.116	ATGCAAATTCAGGGATGGAAAGAACTG|ttggtgttcgtctttgtaaatgaat-- --gaagcaggtctgtatgggacaagcttg|AAGTACACGTGCGTTCATTTTTCCC	-

EXT2	279	41.025	Proximal: 44.130.050 Distal: 44.171.074	TTTGGCCATTCTAATAGATATGTAT|ttgtatcttattgctgttttaattt-- --gc**tattca**tatctatacataagggg|GACTGATAAAACAGGCC**TGAGTC**AT	TATTCA: Deletion hotspot consensus sequence (complement) TGAGTC: Deletion hotspot consensus sequence

The positions of the proximal and distal breakpoints or breakpoint regions were given according to the UCSC Human Genome Browser assembly from March 2006 (NCBI36/hg18) on the respective chromosomes (the reverse strand of chromosome 8 for *EXT1 *and the forward strand of chromosome 11 for *EXT2*). Breakpoint sequences were given, including 25 bp down-and upstream from the breakpoints or breakpoint regions if homologous sequences (marked in grey) were present at the breakpoints. Down-and upstream borders of the deleted sequences are indicated in small characters. Alternating purine-pyrimidine sequences are underlined. Finally, DNA sequence motifs associated with deletions are marked in bold, while the motif sequence and the deletion-associated motif type can be found in the last column.

### In silico analysis of deletion breakpoints

An overview on all results on the presence of microhomologies at the breakpoint junctions, alternating purine-pyrimidine sequences and deletion-associated DNA sequence motifs can be found in Table 2. Analysis of the AT-percentages for the sequences located 125 bp down-& upstream of proximal and distal breakpoints showed AT-enriched regions (≥65%) on both breakpoints for families 338 and 279.

**Figure 2:**
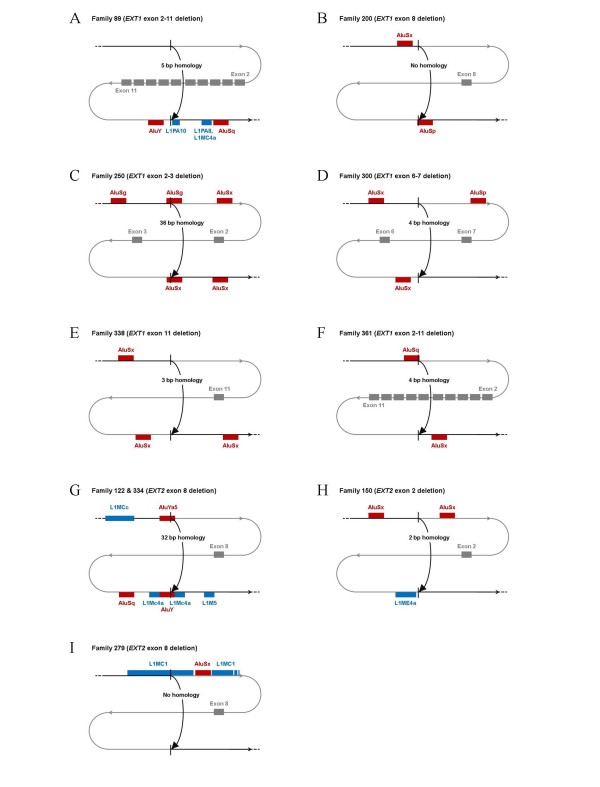
**Schematic representation on all deletions**. The curved line represents the DNA sequence, with in black the non-deleted sequence and in grey the deleted part, including the deleted exons that are represented by grey blocks. The upper and lower horizontal lines represent the 20 kb regions around each deletion breakpoint and are to scale. The middle line, including the deleted exons, is not to scale. Vertical black lines point at the deletion breakpoints. Alu-elements are indicated by red bars, LINE1-elements by blue bars. Sequence homology at the deletions breakpoints is mentioned in the arrow, representing the deletion. A.) Family 89 (EXT1 exon 2-11 deletion), B.) Family 200 (EXT1 exon 8 deletion), C.) Family 250 (EXT1 exon 2-3 deletion), D.) Family 300 (EXT1 exon 6-7 deletion), E.) Family 338 (EXT1 exon 11 deletion), F.) Family 361 (EXT1 exon 2-11 deletion), G.) Families 122 & 334 (EXT2 exon 8 deletion), H.) Family 150 (EXT2 exon 2 deletion), I.) Family 279 (EXT2 exon 8 deletion).

A schematic illustration on all recombination events can be found in Figure 2, including the BLAST2 results concerning homologous regions as well as the UCSC results concerning the presence of *Alu*-and LINE1-elements. Only the 2 kb regions around the breakpoints are represented. No other homologies that could have played a role in the different recombination events were identified outside these regions.

## Discussion

The most prominent DNA-repair mechanism in human cells is NHEJ, involving simple ligation of any two DSB's in the absence of extensive sequence homology. NHEJ is divided into two sub-pathways, classical and non-classical. In classical NHEJ, end-resection is very limited since this pathway only efficiently joins DSB's with overhangs of fewer than four bases. Typical for this mutation mechanism is that it can be facilitated by terminal microhomologies (1-4 bp), although their presence is not necessary [24]. In non-classical NHEJ or "microhomology-mediated end joining" (MMEJ) however, a rare end-joining event takes place which uses longer microhomology regions (5-25 bases) [25]. Non-classical NHEJ can also repair ends of a single DSB in a way similar to SSA, resulting in the generation of small-scale deletions [26].

MMRDR comprises all replication-based mutational models that are predicated upon the use of microhomology for strand misaligning. NHEJ-compatible events involving microhomologies can be explained either by NHEJ or by MMRDR, implicating that both mechanisms do not necessarily rule each other out [17].

Homologous recombination is the second major repair pathway for DSB's. It uses a homologous sequence as a template for repair. However, erroneous recombination on a non-allelic homologous site can lead to chromosomal rearrangements, including deletions. The different submechanisms depend on the homologous sequence that is used. NAHR or "unequal HR" represents the main homologous recombination mechanism. It is the typical mechanism for recurrent deletions and is the most common mechanism underlying disease-associated genomic rearrangements. It occurs between two non-allelic homologous sequences, generally with a length of at least 200 bp, often consisting of repetitive elements such as long or short interspersed nuclear element (LINE's or SINE's including *Alu*-elements) or low copy repeats (LCR's). A second HR-mechanism is SSA, which can take place when DSB's are flanked by direct repeats. This pathway uses these repeat sequences as the identical sequences that HR needs for repair, instead of requiring the presence of a homologous sequence. After 5'-end resection, the 3'-tails simply anneal to each other before one of the 3'-tails can find and base-pair with a homologous sequence. SSA results in simple rearrangements with deletion of the DNA-fragment located between the repeats as well as one of both repeats. Since the success rate of this pathway is inversely related to the distance separating the direct repeats (SSA depends on the formation of a short hairpin loop between breakpoint ends), SSA only accounts for small-scale deletions [27,28].

So, it is clear that gross genetic deletions do not appear randomly in the genome. They are associated with DNA-sequences promoting either one of the above mentioned mutation mechanisms. Additional features that have been shown to play a role in the appearance of large deletions, are sequences rich in adenine and thymine (AT-enriched sequences), alternating purine-pyrimidine repeats and recombination-associated motifs such as translin binding sites, immunoglobulin heavy chain class switch sites and deletion hotspot consensus sequences [23].

To date, single or multi-exon rearrangements in *EXT1 *or *EXT2 *have been routinely screened for by Multiplex Ligation-dependent Probe Amplification (MLPA) [16]. However, since this diagnostic technique does not provide insight into the developmental mechanism of these rearrangements, we characterized for the first time the large genomic deletions in *EXT1 *and *EXT2 *in index patients from ten unrelated families using sequencing analysis and hypothesized on the developmental mechanism of these rearrangements.

Recent studies have demonstrated that high content of *Alu*-elements results in increased frequency of gene disruption by large deletions in several human diseases [29]. *Alu*-elements are by far the most abundant short interspersed nuclear element (SINE's), with an estimated copy number of ~1.4 million [30,31]. They are conserved repeats with a consensus sequence of ~300 bp that have been amplified in primate genomes through retroposition [32] and consist of different subfamilies (AluY, AluSx, ...) (reviewed in [33]). These elements have been proposed to have a number of functions in the human genome, but it is certain that they did have a major impact on genomic architecture, since dispersion of the *Alu*-sequences throughout the genome offers many opportunities for NAHR [34,35], leading to *Alu*-recombination mediated deletion (ARMD)-events [36,37]. LINE1-elements, a subgroup of the long interspersed nuclear elements (LINE's) are also known to frequently provide sequence homology for NAHR. Consequently, we analyzed the whole genomic sequences of *EXT1 *and *EXT2 *for the presence of these elements. The *EXT1 *genomic region was found to be enriched in *Alu*-elements (13.4% compared to 10.6% in the human genome), while the *EXT2 *region showed to be rich in LINE1-elements (20.4% compared to 16.9% in the human genome) [38]. This might explain why both genes are more prone to deletions.

In the *EXT1 *exon 2-3 deletion of family 250 as well as in the *EXT2 *exon 8 deletion of unrelated families 122 (Spain, familial MO) and 334 (Bulgaria, de novo MO) extensive sequence similarity was identified between the breakpoint regions. These rearrangements were shown to be caused by recombination between Alu-repeats, giving rise to the formation of a novel complete recombinant Alu-sequence. We concluded these deletions to be caused by NAHR, with this hypothesis further supported by the fact that the *EXT2 *exon 8 deletion was recurrent. Additionally, these deletion-causing recombinations might have been facilitated by the presence of multiple deletion-associated DNA sequence motifs.

For family 200 and 279, no homology was found at the breakpoints of the respective *EXT1 *exon 8 deletion and the *EXT2 *exon 8, although multiple LINE1-and *Alu*-elements were identified in the proximity of the breakpoints as well as multiple deletion-associated DNA sequence motifs. Consequently, these cases were found to be consistent with the classical NHEJ mutation mechanism.

For family 89, the breakpoints of the *EXT1 *exon 2-11 deletion were located within a 5-bp homologous sequence. Multiple LINE1-and *Alu*-elements were identified at the distal breakpoint, while the proximal breakpoint only showed the presence of the complement of a deletion hotspot consensus sequence. The 5 bp-microhomology at the breakpoint junctions implied that this deletion was probably caused by non-classical NHEJ or by MMRDR.

For the remaining 4 families (Family 300 with *EXT1 *exon 6-7 deletion, Family 338 with *EXT1 *exon 11 deletion, Family 361 with *EXT1 *exon 2-11 deletion, Family 150 with *EXT2 *exon 2 deletion), deletion breakpoints were located within microhomologies (2-4 bp), consistent with either the classical NHEJ or the MMRDR mechanism. *Alu*-elements were found in the proximity of all breakpoint regions, except for the distal breakpoint of the *EXT2 *exon 2 deletion in family 150, while deletion-associated motifs were found at the proximal breakpoint of the *EXT1 *exon 2-11 deletion from family 362.

In our series, only two patients harbored an identical deletion (*EXT2 *- exon 8 deletion). In MO, the size and location of the various intragenic EXT deletions does not seem to correlate with phenotypical differences as all these deletions are assumed to cause loss-of-function of the respective tumor suppressor gene *EXT1 *or *EXT2*. The same is true for EXT loss of function point mutations, where no intragenic genotype-phenotype correlation is observed [16]. The only correlation that can be observed is for larger deletions causing the contiguous gene syndromes Langer Giedion syndrome [19] and Proximal 11p Deletion syndrome (Potocki-Shafer syndrome) [20,21], but such patients were not included in our series. Furthermore, our patient dataset was too small to confirm previous observations of EXT1 patients being more severely affected compared to EXT2 patients [16].

The identification of the breakpoints of this subset of patients makes it possible to design allele specific PCR-assays allowing targeted screening for recurrent deletions. Performing these allele specific PCR-amplifications on a larger set of EXT-deletion patients can further confirm the absence or presence of deletion hot spots for MO-causing single-or multi-exon deletions in *EXT1 *and *EXT2*.

## Conclusions

Out of the ten deletions analyzed in this study, NAHR was found to be the causal mechanism in two cases. Two deletions were caused by classical NHEJ, while four other rearrangement events could be explained by either classical NHEJ or MMRDR. Finally, non-classical NHEJ or MMRDR were the possible mutation mechanisms for the last deletion. One deletion, typically caused by NAHR, was shown to be recurrent in 2 patients, but no clear deletion breakpoint hotspots could be identified within our set of patients.

So, we can conclude that MO-causing large genomic deletions in *EXT1 *or *EXT2 *are caused by a variety of mutation mechanisms, emphasizing once more the huge genetic variability for MO.

## Competing interests

The authors declare that they have no competing interests.

## Authors' contributions

IJ: performed mutation studies, drafted the manuscript, performed breakpoint characterization studies

DdJ: designed the array, analyzed the results

KM: performed mutation studies, performed breakpoint characterization studies

PCWH: designed the array, commented on the manuscript

KS: designed the array, analyzed the array data

WW: designed the study, interpreted the results, reviewed manuscript

All authors have read and approved the final manuscript.

## Pre-publication history

The pre-publication history for this paper can be accessed here:

http://www.biomedcentral.com/1471-2350/12/85/prepub
